# Development and psychometric testing of quality nursing care scale in Mongolia

**DOI:** 10.1186/s12912-021-00586-3

**Published:** 2021-04-28

**Authors:** Basbish Tsogbadrakh, Wipada Kunaviktikul, Thitinut Akkadechanunt, Orn-Anong Wichaikhum, Khulan Gaalan, Oyungoo Badamdorj, Azadeh Stark

**Affiliations:** 1 School of Nursing, Mongolian National University of Medical Sciences, Zorig Street, Ulaanbaatar, 14210 Mongolia; 2 Faculty of Nursing, Chiang Mai University, Chiang Mai, Thailand; 3 Department of International Cyber Education, Graduate School, Mongolian National University of Medical Sciences, Ulaanbaatar, Mongolia; 4 Department of Pathology and Laboratory Medicine, Henry Ford Health System, Detroit, MI USA; 5 School of Interdisciplinary Studies, University of Texas at Dallas, Richardson, TX USA

**Keywords:** Quality nursing care, Measurement, Instrument development, Mongolia

## Abstract

**Background:**

Quality Nursing Care (QNC) is fundamental to the profession of nursing practice. Perception of QNC differ across the globe because of differences in social norms, cultural values and political ambiance and economy. This study aimed to develop a QNC instrument congruent with the Mongolian (QNCS-M) healthcare system and cultural values and societal norms.

**Methods:**

Exploratory sequential mixed-method design was implemented to develop and assess performance of QNCS-M. First, we focused on developing the components of QNCS-M and their operational definitions. Second, we dedicated to ascertaining psychometric performance of QNCS-M. The field testing consisted of assessing the construct validity and internal consistency reliability. Correlation between QNCS-M and the criterion tool, Quality of Nursing Care Questionnaire-Registered Nurse was evaluated.

**Results:**

The initial version of QNCS-M contained 66 items of which 7 (I-CVI < .78) were deleted after item-content validity assessment. The total-item correlation analysis yielded to exclusion of another 3 items (<.3). Additional 12 items were excluded after inter-item correlation (<.3, >.7). Results from Spearman rank-order correlation analysis of the remaining 44 items indicated relationship between social desirability and 6 items (*r* = −.09 to *r* = .11). These items were excluded to reduce the likelihood of potential information bias. A total of 38 items remained for exploratory factor analysis. Results from exploratory factor analysis yielded eigenvalues > 1.0 for the 9 domains. Three domains contained items fewer than 3. These domains and 2 items (factor loading <.4) were eliminated, yielding to 6 domains with 36-item. Results from internal consistency reliability yielded an overall Cronbach’s α = .92; the coefficient values for the 6 domains ranging between .72 and .85 and Pearson correlation for stability reliability yielded an acceptable (*r* = .82, *P* < .001).

**Conclusion:**

Improving the quality of healthcare services delivered by nurses is a priority for the Mongolian government. The development of QNCS-M is a major stride in addressing this concern. The final version of QNCS-M which contains 36 items, loaded into 6 domains, was morphed to the specifics of the Mongolian healthcare systems and cultural values and societal norms. QNCS-M demonstrates a high level of content and construct validity with acceptable reliability.

**Supplementary Information:**

The online version contains supplementary material available at 10.1186/s12912-021-00586-3.

## Background

Provision of quality of healthcare services is the tenet of success in patient outcomes. The importance of quality of healthcare services in the nursing profession and by the nursing professional has been addressed since the time of Florence Nightingale [1]. In more recent times, the nursing scholars and the nursing administrators and leaders have brought the concept of Quality Nursing Care (QNC) to the frontline of the nursing profession. Several milepost achievements such as establishment of the National Database of Nursing Quality Indictors by the American Nursing Association (ANA) in 1998 or the formation of the Task and Finish Group for a rapid appraisal of the evidence on nursing measures in April of 2012, at the behest of the Chief Nursing Officer, by the UK National Health Services underline the prominence of QNC in the nursing profession [2, 3]. The indicators for QNC developed by the ANA or by the UK National Health Services, can be used as guidelines for the nursing professionals in other countries, but their applications might not totally be relevant and/or applicable to countries such as Mongolia. For example, some of the proposed QNC indicators are abstruse and difficult to either utilize and/or measure. This argument is supported by the extent of publications, by the nursing professionals across the globe, addressing QNC in various healthcare settings [4–6].

Delivery healthcare services by the nursing professionals is complex. This complexity arises from the wide range of healthcare services, physical, psychological, emotional, social and spiritual care, delivered by the nursing professionals [7]. Additionally, the diversity in patients’ needs adds another layer of complexity to the delivery of healthcare services and to the concept of QNC [1, 7, 8]. Moreover, variations in cultural and social norms and political ambiances across the globe adds another tier of complexity to the delivery of healthcare services by the nursing professionals and to the concept of QNC. Finally, QNC is a subjective perception, at least in some of the domains of nursing care. This subjectivity can be influenced by personal experiences and social and cultural norms and values of patients and/or the nursing management [9–11]. Therefore, application of a universal set of quality indicators for assessment of QNC in different countries can not be justified; because this application potentially can lend itself to spurious and deleterious results. We report here the development and field testing of an instrument to assess the psychometric properties of the Quality Nursing Care Scale (QNCS) in Mongolia, hereafter, referred to as QNCS-M.

## Methods

### Research design and approach

We implemented a two-phase exploratory sequential mixed-method design to develop and validate QNCS-M. During the phase I of this project, we dedicated time and effort to develop an instrument merited on the 5-step proposed by DeVellis [12] while, phase II was dedicated to ascertaining psychometric performance of QNCS-M.

### Phase I: instrument design and development

This phase began by drafting the blue-print of QNCS-M which was based on the overall concept of QNC, first proposed by Kunaviktikul [7]. However, the components of QNCS-M and its 5 dimensions were established based on the results of a previously implemented qualitative study in Mongolia and conducting a comprehensive literature review [13]. We then proceeded with establishing the operational definition of each of the five dimensions of QNCS-M. These operational definitions were unanimously agreed by the members of our research team. We defined the Physical Care as the interventions provided by the nursing professionals with the objective of symptom management and assistance with activities of daily life. The operational definition of Psychological Care was focused on activities provided by nursing professionals that encourage patients in assuming self-directed activities to relieve mental distress. We defined the operational operation of Emotional Care as supports and activities provided by the nursing professionals to understand and to support the frame of minds and emotional states of patients because of their illness. We explicated the operational definition of Social Care as the process of building positive social relationships with the patients and creating a positive and supportive ambiance to prevent depression among patients due to their sense of isolation. Finally, the operational of definition of Spiritual Care was established as recognition of and respect for patients’ religious beliefs and proclivity. In addition, we included the practice of acceptance and respect for patients’ cultural diversity by the nursing professionals in the operational definition of Spiritual Care. This step was followed by drafting and scaling of a total of 66 items in English language. Each item was scaled based on a five-point Likert scale, ranging 0–4. The sum of scores reflected the level of quality of nursing care with value of 0 indicating the lowest quality of nursing healthcare services, while the value of 264 indicated the highest possible level.

The English translated version of QNCS-M was reviewed and evaluated by a panel of seven academicians from US, Thailand and Mongolia with expertise in instrument development and content validity assessment. Per the recommendations of the panel, seven questions were deleted because the item-content validity index (I-CVI) < .78 [14]. Item-content validity indices (I-CVIs) of the remaining items ranged from 0.85 to 1.0. The revised version of QNCS-M was translated back to Khalkha dialect and was reviewed by a panel of 10 nursing academicians and directors in Mongolia. The panel assessed our instrument for its clarity, readability, accuracy and its length. The panel of reviewers flagged 12 items as ambiguous. These 12 items were revised and restructured to ascertain clarity in Khalkha dialect. The latest version of this 59-item instrument was pretested among 20 nursing professionals working in the State Central Third Hospital in Mongolia. The Cronbach’s alpha coefficients of the instrument was .94.

### Phase II: instrument psychometric performance

**Fig. 1:**
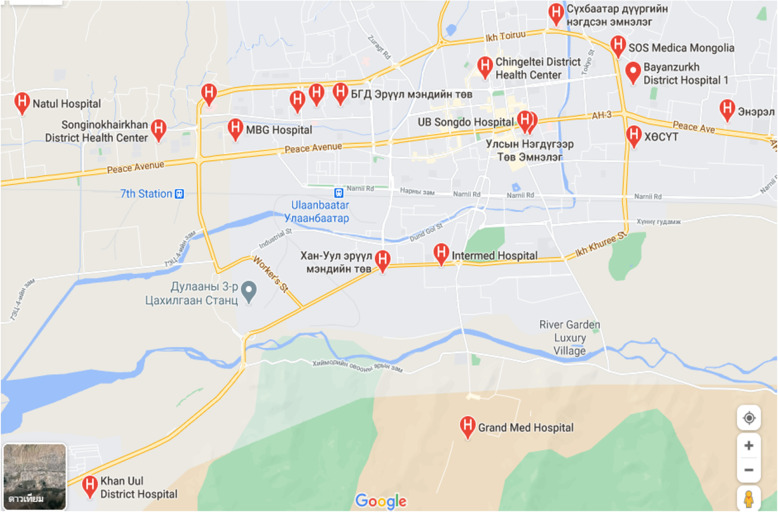
Location of the Participating Hospitals in the City of Ulaanbaatar, Mongolia

Assessment of the psychometric performance of QNCS-M consisted of two steps. During step one, we focused on field testing of the 59-item instrument for its construct validity and internal consistency reliability. Field Testing of Instrument was conducted in nine public hospitals in city of Ulaanbaatar. These hospitals with 984 strong nursing professionals are secondary and tertiary level hospitals and serve as the referral system for the entire country [15]. A total of 485 nursing professionals using multi-stage sampling technique were randomly identified and recruited to take part in our study [16]. First, we applied a proportional random sampling approach to determine the number of participants; our sampling approach was adjusted for the size of the nursing staff in each hospital. In the second step, proportionate stratified sampling method was used to select the number of nurses from different departments in each hospital. Finally, prospective study participants randomly were selected from the name list of each nursing department. Only those who met the eligibility criteria were recruited to participate in our study. We have outlined location of the participating hospitals in the city of Ulaanbaatar, Mongolia (Fig. 1, Additional file 1: Appendix A). Additionally, the list of hospitals and percentage of sample size from each hospital are provided in Additional file 1: Appendix A.

In calculating the total sample size, we assumed participant-to-item ratio of 7:1 [17], we further inflated our sample size by 18% to account for the sample attrition due to no-response. This approach was justified based on previous reports suggesting a sample size of 300–500 as adequate for factor analysis procedure [12, 18]; additionally, the relatively small number of the nursing professionals (*n* = 985) working in the nine public hospitals was another justification for our sample size calculation.

The nursing professionals were eligible to participate in our study if: A) They were actively employed by the public hospital system in the city of Ulaanbaatar; B) They were performing their professional duties in one of the nine accessible secondary or tertiary care hospitals in Mongolia; C) They had at least 1 year of work experience in direct patient-care health services and D) They were willing to participate in our study. Nurses who had participated in the pre-testing stage were not eligible to participate. This restriction was imposed to reduce the likelihood of information bias.

A trained research assistant introduced the objective of the study to the prospective study participants and obtained their signed informed consent forms. The research assistant then distributed copies of our 59-item QNCS-M and Marlowe-Crowne Social Desirability Scale (MCSDS) and Demographic questionnaires to the study participants and instructed them to return the completed instruments within 2-weeks in sealed envelopes in the drop-boxes which were placed in the Nursing Departments of the participating hospitals. Returned surveys were anonymous. This section of our study started in February of 2019 and was completed in December 2019.

### Statistical analysis

We used descriptive statistics to summarize the demographic characteristics of the study participants and distributions of responses to each item listed in our 59-item instrument. Inter-item correlation was examined by using the Spearman rank-order correlation. Item-total correlation was calculated by using reliability analysis. The criterion for selecting qualified items to constitute a consistent scale included item-total correlation and a corrected item-subscale correlation of .30 or higher [18]. Inter-item correlations ranged from .30 to .70 [19]. The internal consistency reliability was analyzed using Cronbach’s alpha coefficient. A reliability coefficient above .70 is considered acceptable for a new scale [12]. Finally, we applied Spearman rank-order correlation used to assess the relationship between MCSDS score and each item of QNCS-M.

We then proceeded with calculating the Kaiser-Meyer-Olkin (KMO) value to ascertain the adequacy of our sample size. Additionally, we performed Bartlett’s test of sphericity to evaluate for item redundancy of QNCS-M [20]. The criteria for determining the factor solution of factor extraction included: 1) a factor with an eigenvalue of 1.00 or above, 2) item with a factor loading of .40 or above, 3) no factor with fewer than three items [21]. Finally, we performed exploratory factor analysis (EFA) to assess construct validity of QNCS-M. This approach permitted us to investigate if the internal structure of the scale of QNCS-M fit with the original theoretical definition and conceptual framework [7].

### Step 2. Performance comparison against QNCQ-RN, the criterion tool

In this step, we assessed the empirical relationship between QNCS-M and the criterion tool, the Quality of Nursing Care Questionnaire-Registered Nurse (QNCQ-RN) [22]. The QNCQ-RN has been widely to assess quality nursing care instruments in hospital settings [23, 24]. Permission was obtained from the authors; QNCQ-RN was translated into Khalkha dialect and then was translated back to English to ascertain the validity of the translated version.

We recruited 54 nursing professionals from State Central First Hospital, Bayangol District Health Center and Bayanzurkh District General Hospital. We imposed three inclusion criteria:1) Providing healthcare services in the role of a nursing professional and 2) Having at least 1 year of work experience in direct patient-care health services and 3) Willingness to participate in our study. The sample size of 54 was based on the expected correlation coefficient value of 0.4, power of 80% and, alpha = .05; our calculation yielded a minimum sample size of 46 [25]; however, we inflated our sample size by 18% to account for non-responders.

The lead investigator introduced the objective of this segment of our study and copies of QNCS-M and the QNCQ-RN were distributed among the study participants. The study participants were instructed to return the completed instruments within 2-weeks in sealed envelopes in the drop-boxes which were placed in the Nursing Departments of the two participating hospitals. All the 54 participants returned their anonymous completed questionnaires; the returned questionnaires were evaluated by the lead investigator for completeness; data were entered into the databases for statistical analysis. To assess the validity scale of QNCS-M, we calculate the correlation between QNCS-M and the QNCQ-RN using the Spearman rank-order statistical technique [26]. Our calculation yielded a statistically significant correlation between the two instruments (*r* = .28, *P* < .03).

### Ethical considerations

This study was approved by the Research Ethics Committee of the Faculty of Nursing, Chiang Mai University (Approval-020/2019), the Bio-Medical Research Ethics Committee of Mongolian National University of Medical Sciences, and the participating hospitals in Mongolia. Informed written consents were obtained from all participants. Confidentiality of study participants were respected by the research team. All personal identifiers were deleted after the final data quality and assurance. Data were stored in a password protected database behind the IT firewall of the Mongolian National University of Medical Sciences.

## Results

### Instrument psychometric performance

Of the 485 study participants, 456 (94%) returned their questionnaires; we excluded 16 (3.2%) of the returned questionnaires because of missing information. Questionnaires from a total of 440 (90.7%) participants were complete and deemed as acceptable for further analysis. The mean age of study participants was 36 years (SD = 10; range 22–59). The majority of the nurses were female (*n* = 431, 98.0%), and more than half of the subjects were married (*n* = 313, 71.1%). More than half of the nursing professionals (*n* = 262, 59.5%) who contributed to our study, had earned a bachelor’s degree in nursing science (Table 1).

**Table 1:** Demographic Characteristics of the Nurses (*n* = 440)

Demographic Characteristics	Frequency	Percentage (%)
Age (years) ($\overline{\mathrm{X}}$ = 36.36, SD = 10.06, Range = 22–59)		
20–29	162	36.8
30–39	97	22.0
40–49	128	29.1
50–59	53	12.0
Gender		
Female	431	98.0
Male	9	2.0
Marital status		
Single	98	22.3
Married	313	71.1
Divorced	11	2.5
Widowed	11	2.5
Separated	7	1.6
Educational level		
Diploma	163	37.0
Bachelor	262	59.5
Master or above	15	3.4
Work experience (years)		
1–5	165	37.5
6–10	74	16.8
11–15	50	11.4
More than 15	151	34.3
Work department		
Surgical nursing	75	17.0
Medical nursing	365	83.0
Hospital		
State Central First Hospital	145	33.0
State Central Second Hospital	60	13.6
State Central Third Hospital	119	27.0
Khan Uul District General Hospital	18	4.1
Songinokhairkhan District General hospital	20	4.5
Bayanzurkh District General Hospital	21	4.8
Chingeltei District Health Center	19	4.3
Bayangol District Health Center	18	4.1
Sukhbaatar District Health Center	20	4.5

#### Item analysis, item-total correlation and inter-item correlation of QNCS-M

Results from the item analysis, yielded item mean values ranging from 2.57 to 4.64 (SD = .57–2.01). Findings from the total-item correlation analysis yielded coefficient values ranging between .25 to .64. For three items, #5 (*r* = .26), # 18 (*r* = .29) and # 42 (*r* = .25), the total-item correlation coefficient values were less than .3 [18]; therefore, we decided against including these three items in the revised version of QNCS-M. A total of 56 items comprised the latest version of QNCS-M. Findings from inter-item correlation were assessed and coefficient values between a minimum value of .30 and a maximum value of .7 were considered as acceptable [19]. Our calculation yielded correlation coefficient values for 12 items below .3. These 12 items were removed which reduced the number of items in QNSC-M from 56 to 44 items. (Additional file 1: Appendix B).

Results from the Spearman rank-order correlation analysis of the remaining 44 items, indicated relationship between MCSDS and 6 items, #Q11 (*r* = .11, *p* = .02), #Q20 (*r* = −.10, *p* = .03), #Q31 (*r* = .09, *p* = .05), #Q35 (*r* = .10, *p* = .02), #Q56 (*r* = .10, *p* = .03) and #Q57 (*r* = −.09, *p* = .03). We decided to exclude these items to reduce the likelihood of potential information bias. A total of 38 items remained for exploratory factor analysis. (Additional file 1: Appendix B).

#### Exploratory factor analysis

Results from Kaiser-Meyer-Olkin measure of sampling adequacy yielded a value of .90, which indicates a strong support for the adequacy of items (*n* = 38) for factor analysis [21]; furthermore, results from Bartlett’s test of sphericity (X_i_^2^ = 7021.82, *P* = .0001) supported that the items were in a linear relationship [19]. Therefore, we proceeded with performing exploratory factor analysis (EFA). Our EFA analysis of the 38 items, yielded eigenvalues greater than 1.0 for a total of 9 domains. However, upon further assessment of the results of EFA, three domains (factors, 7–9) contained items fewer than the minimum requirements of 3 items; thereby, we eliminated these domains [21].

Results from scree plot factor analysis showed six domains above the elbow of the curve. Therefore, we decided to retain the six domains within the overall structure of the QNCS-M. These six domains were labeled as: “Independent Nursing Role”, “Inter-dependent Nursing Role”, “Psychological Element”, “Social Milieu”, “Personal Milieu” and “Spiritual Force”. Two items, # 27 and 36, in the 38 QNCS-M showed no loading (coefficient < .4) to any of the six domains. These two items (#27 and 36) were excluded, yielding to a 36-item QNCS-M. A second EFA was conducted to ascertain loading adequacy of these 36 items. Results indicated that these 36 items maintained the loading values greater than .40. These six domains explained 55.41% of the total variance with eigenvalues ranging between 9.86 to 1.17 (Table 2).

**Table 2:** Item descriptions, item-total correlations, Cronbach’s alpha and factor loadings (*n* = 440)

Item No.	Description	Corrected item-total correlation	Cronbach alpha	Factor loadings					
				F1	F2	F3	F4	F5	F6
Overall QNCS-M			0.92	Total explained variance = 55.41%					
**Interdependent Nursing Role**			0.74	Eigenvalue: 1.17; Variance:3.25					
4	Interventions to relieve patients’ physical suffering.	0.41		.75					
6	Prompt care when I notice patients’ clinical symptoms.	0.35		.73					
2	Recognize clinical symptoms	0.33		.69					
3	Priority is concern for relieving or reducing physical suffering.	0.37		.63					
**Independent Nursing Role**			0.72	Eigenvalue:1.63; Variance:4.53					
12	Help patients to maintain their hygiene	0.38			.64				
10	Determine whether or not my patients are getting enough sleep or rest	0.38			.63				
9	Adequate daily exercise programs or physical activities	0.41			.58				
7	Adequate diet for all patients to regard individual needs for healing.	0.46			.53				
8	Adequate care to solve the process of elimination problems	0.47			.52				
**Psychological element**			0.83	Eigenvalue:2.14; Variance: 5.96					
16	encourage and allow time for patients to talk about their priority concerns.	0.55				.70			
13	spend enough time with patients to sincerely discuss their feelings.	0.50				.67			
17	encourage patients’ self-confidence to assist in maintaining their health and help manage their illnesses.	0.54				.64			
15	encourage the patient to be resolute and determine to get better.	0.49				.58			
14	say inspiring words to my patients. e.g., your treatment is working well; you’re getting better day by day.	0.51				.53			
21	educate each patient specifically for individual needs.	0.53				.51			
22	give up-to-date and evidence-based health education for patients.	0.48				.48			
**Personal Milieu**			0.84	Eigenvalue: 3.73; Variance: 10.36					
30	Available when patients call me or ring their bell.	0.43					.77		
29	Have ability to apologize to patients if i make mistakes.	0.39					.73		
27	Sincere listen to what my patients is telling me.	0.45					.73		
28	Patient and tolerant with my patients and their families.	0.53					.69		
26	Offer a personal greeting to patients that i encounter when i arrive at work.	0.43					.65		
36	Allow my patients to do things that get them to calm down.	0.47					.63		
34	Softly touch my patients’ shoulders or hands when appropriate.	0.58					.61		
33	Allow time to make an effort to cheer my patients up.	0.66					.58		
38	Make patients feel welcome on the ward	0.52					.57		
**Social Milieu**			0.78	Eigenvalue: 1.40; Variance: 3.89					
46	I maintain an environment that promotes healing e.g., quiet, clean and good ventilation.	0.56						.72	
45	I draw a curtain to separate patients from others when performing any physical care procedures.	0.39						.72	
47	I ensure the promotion of safety and security of all patients on the ward.	0.49						.69	
48	I ensure a pleasant atmosphere all the time whenever possible.	0.53						.64	
**Spiritual Force**			0.85	Eigenvalue:9.86; Variance:27.40					
52	I’m not partial in regard to patients practice of traditional beliefs	0.37							.80
51	I maintain consideration for patients’ beliefs	0.49							.79
50	I volunteer to help when patients and their families desire to perform religious activities in the unit	0.51							.73
59	I agree that all patients from different cultural backgrounds should be able to choose their own individually preferred benefits	0.38							.67
49	I provide the opportunity for religious activities in the unit.	0.47							.64
54	I consider the patients’ different health-related attributes and cultural needs when I develop nursing care plans.	0.54							.61
55	I freely discuss with patients about any restrictions relating to their cultures	0.58							.51

#### Reliability assessment and concurrent validity

Results from internal consistency reliability of QNCS-M yielded an overall Cronbach’s alpha coefficient value of .92; while, the coefficient values for the six domains ranged between .72 and .85 (Table 2). Test-retest reliability of the field-tested items was evaluated among 40 volunteers 2 weeks after the completion of field testing. Results from Pearson correlation for test-retest reliability yielded a coefficient value of .82 (*r* = .82, *P* < .001). Finally, we detected a positive and statistically significant correlation (*r* = .28, *P* = .03) between QNCS-M and the QNCQ-RN, the reference criterion. This correlation further lends supporting evidence of the concurrent validity of our instrument.

## Discussion

We implemented a sequential exploratory mixed-method study with the primary objective of developing a valid instrument to assess Quality Nursing Care in Mongolia. The necessity of developing a Quality Nursing Care Scale that targets the Mongolian healthcare system has been a priority for the Mongolian nursing and the other healthcare professionals for some time. Implementation of internationally developed instruments in Mongolian hospitals is not practical primarily because of three reasons. First, health practices in Mongolia, similar to any other nation, are influenced by its history, its culture and its social norms and values. Second, perception of Mongolian patients about healthcare services and quality of these services are influenced by their religious health practices and cultural values about health and well-being. Third, delivery and quality of healthcare services in Mongolia, similar to other nations, are influenced by its political structure and its economy. Therefore, we rest our justification for developing a Quality Nursing Care Scale in Mongolian (QNCS-M) based on the incompatibility of the already developed scales with the Mongolian society [8, 22, 27]. The development of QNCS-M is a major stride in addressing the concerns of the Mongolian healthcare professionals and administration.

The premise of QNCS-M was an extensive based on a previously implemented qualitative research and literature review [13]. However, the final version of QNCS-M, which contains 36 items and loaded into 6 domains, was morphed to the specifics of Mongolian healthcare systems and cultural values about the quality of healthcare services. Initially we attempted to combine the first (interdependent nursing role) and second (independent nursing role) domains because both domains were addressing the concept of physical care. However, findings from our factor analysis suggested strong correlation within each domain, most likely due to the number of items within each factor; therefore, we opted for listing these two domains separately. The QNCS-M is quick to complete, the average completion time is about 15 min, and easy to score.

The QNCS-M was designed to demonstrate conformities with previously developed quality nursing care instruments and yet to address the specifics of the Mongolian healthcare system. For instance, the importance of application of quality of standards and attention to the physical status of patients have been reported as important in creating positive perceptions about the quality of healthcare services [28]. Several previously developed Quality of Nursing Care Scales have allocated one domain, labeled as Physical Care, to assess the quality of healthcare services delivered by the nursing professionals [8, 22, 29]. However, in designing and developing QNCS-M, we decided to allocate two domains, Independent Nursing Role and Interdependent Nursing Role, to assess the quality of healthcare services delivered by Mongolian nursing professionals. We justified our decision based on the adequacy of numbers of the items and the strong correlation among the items within each domain. But equally important, our decision was based on the independent role of the Mongolian nursing professionals in the delivery of healthcare services that are classified under the domain of Independent Nursing Role. The Mongolian nursing health professionals are the sole responsible entities in decision making and delivery of nursing care such as feeding, toileting, bathing, transferring and dressing. While, the Inter-dependent Nursing Role emphasizes the collective approach and inter-dependency of healthcare professionals in assessing patients’ health status, delivering healthcare services and managing their health conditions.

Limited or no concern from the nursing professionals about patients’ psychological wellbeing, i.e., limited patient-communication, can negatively affect perception of quality of healthcare services delivered by the nursing professionals [28]. The importance of patients’ psychological states in the delivery of healthcare services, provision of information and/or assessing patients’ physical well-being have been well documented, emphasized and applied in scales such as GNCS [8, 22, 27, 29]. In consequence, we designed and developed QNCS-M to capture the most significant aspect of psychological needs and psychological care that are congruent with Mongolian cultural health values and healthcare practices. For example, QNCS-M contains items that address the nursing professionals’ responsibilities for psychological state of patients or their roles in inspiring patients to recovery and boosting their confidence in assuming and maintaining self-care.

Supportive environment, human interaction and religiosity/spirituality are important components of quality of healthcare services; furthermore, these attributes of a healthcare system which reflect the quality of services can accelerate the recovery rate and improve overall health of patients [30, 31]. Under the domain of Social Milieu, the focus of quality of nursing care is on the efforts and attention of the nursing professionals in creating a social environment for patients to promote patients’ safety and to ascertain their security and privacy. In the domain of Personal Milieu, the focus of quality of nursing care is on the efforts and attention of the nursing professionals on establishing constructive communication and “human touch” with their patients. The domain of Personal Milieu of QNCS-M is comparable to either psychological or emotional domains of previously developed scales [8, 22, 27, 29]. Finally, in the domain of Spiritual Force of the QNCS-M the focus of quality of nursing care pivots on the awareness of the nursing professionals about their patients’ religious affiliation. Additionally, attention has been given to the nursing professionals’ respect and support for their patients’ desires to perform their religious rituals and religious cultural inclination, i.e., use of religious healers or observance of dietary restrictions. The QNSC-M has dedicated one full domain to the concept of religiosity/spirituality and the quality of nursing care. This is in contrast to previously published scales, in which the concept of religiosity/spirituality is embedded within the domains of emotional care or psychological domain [22, 27, 29].

## Conclusion

QNCS-M is a 6-domain, 36-item instrument designed and developed to assess the perception of quality of healthcare services delivered by the nursing professionals in publicly owned and operated hospital systems in Mongolia. QNCS-M demonstrates a high level of content and construct validity with acceptable reliability. The QNCS-M is quick to complete, the average completion time is about 15 min, and easy to score.

The development of QNCS-M is a major stride in addressing the concerns of the Mongolian healthcare professionals and administration.

### Strengths and limitations

The main strength of our study is its design approach which involved both the nursing professionals and patients in generation of items. Our study has two limitations. First, the instrument was validated only by the nursing professionals. Second the study was implemented in the hospital systems, owned and operated by the Mongolian government. Therefore, its applicability might be limited to other settings such as private for profit or not-for-private hospitals.

### Implications

QNCS-M is the segue to improvement of quality of healthcare services delivered by the nursing professionals in Mongolia. Although QNCS-M was designed and developed for application in Mongolian society, this newly developed instrument can be utilized as a guideline in other countries with healthcare systems similar to Mongolia. Finally, QNCS-M can be used in academic nursing focusing as an educational tool in teaching the concept of quality nursing healthcare services.

## Supplementary Information

**Additional file 1: Appendix A**. Number of Study Participants Stratified by Participating Hospital. **Appendix B**. The Final Version of QNCS-M was shortened from the 66 to 36 items

## Data Availability

The data garnered during the current study and the final dataset used for statistical analysis are available from the corresponding author on reasonable request. The initial (66 item) version and the final version (36 item) of the instrument Quality of Nursing Care Services in Mongolia (QNCS-M) are available from the corresponding author on reasonable request.

## Abbreviations

QNC: Quality Nursing Care
QNCS-M: Quality Nursing Care Scale – Mongolia
QNCQ-RN: Quality Nursing Care Questionnaire – Registered Nurses
MSCDS: Marlowe-Crown Social Desirability Scale
NDNQI: National Database of Nursing Quality Indictors
ANA: American Nursing Association
UK: United Kingdom
I-CVI: Item-content validity index
US: United States
KMO: Kaiser-Meyer-Olkin
EFA: Exploratory Factor Analysis

## References

[1] Burhans LM, Alligood MR (2010). Quality nursing care in the words of nurses. J Adv Nurs.

[2] Montalvo I. The national database of nursing quality indicators (TM)(NDNQI®). Online J Issues Nurs. 2007;12(3). available from: https://search.proquest.com/docview/229585708?pq-origsite=gscholar&fromopenview=true.

[3] Maben J, Morrow E, Ball J, Robert G, Griffiths P. High Quality Care Metrics for Nursing. National Nursing Research Unit, Florence Nightingale School of Nursing and Midwifery, King’s College, London; 2012. available from: https://eprints.soton.ac.uk/346019/1/High-Quality-Care-Metrics-for-Nursing----Nov-2012.pdf.

[4] Maurek-Melnyk B, Gallagher-Ford L, English-Long L, Fineout-Overholt E (2014). The establishment of evidence-competencies for practicing registered nurses and advanced practice nurses patient outcomes, and costs. Worldviews Evid-Based Nurs.

[5] Idvall E, Rooke L, Hamrin E (1997). Quality indicators in clinical nursing: a review of the literature. J Adv Nurs.

[6] Kunaviktikul W, Anders RL, Chontawan R, Nuntasupawat R, Srisuphan W, Pumarporn O, Hanuchareonkul S, Hirunnuj S (2005). Development of indicators to assess the quality of nursing care in Thailand. Nurs Health Sci.

[7] Kunaviktikul W, Anders RL, Srisuphan W, Chontawan R, Nuntasupawat R, Pumarporn O (2001). Development of quality of nursing care in Thailand. J Adv Nurs.

[8] Leino-Kilpi H (1996). Patient as an evaluator of nursing services. Proceedings of the Congress in Nursing Administration.

[9] Radwin L (2000). Oncology patients' perceptions of quality nursing care. Res Nurs Health.

[10] Charalambous A, Beadsmoore A (2009). Quality nursing care: a selective review of the literature of patients’ and nurses’ interpretations. Sci J Hell Regul Body Nurses.

[11] Bogaert PV, Kowalski C, Weeks SM, Heusden DV, Clarker SP (2013). The relationship between nurse practice environment, nurse work characteristics, burnout and job outcome and quality of nursing care: a cross-sectional survey. Int J Nurs Stud.

[12] DeVellis RF (2012). Scale development theory and applications.

[13] Tsogbadrakh B, Kunaviktikul W, Akkadechanunt T, Wichaikhum OA, Turale S (2020). Nurse and patient perceptions of quality nursing Care in Mongolian Public Hospitals. Pac Rim Int J Nurs Res.

[14] Lynn MR (1986). Determination and quantification of content validity. Nurs Res.

[15] Tsilaajav T, Ser-Od E, Baasai B, Byambaa G, Shagdarsuren O, Boyer S (2013). Mongolia health system review. Health Syst Transit.

[16] Burns N, Grove SK (2010). Understanding nursing research: building an evidence-based practice.

[17] Nunnally JC, Bernstein IH (1994). Psychometric theory.

[18] Nunnally JC (1978). Psychometric theory.

[19] Knapp TR, Brown JK (1995). Ten measurement commandments that often should be broken. Res Nurs Health.

[20] Hair JF, Black WC, Babin BJ, Anderson RE, Tatham R (2006). Multivariate data analysis.

[21] Tabachnick BG, Fidell LS, Ullman JB (2007). Using multivariate statistics.

[22] Safford BJ, Schlotfeld RM (1960). Nursing service staffing and quality of nursing care. Nurs Res.

[23] Mrayyan MT (2006). Jordanian nurses’ job satisfaction, patients’ satisfaction and quality of nursing care. Int Nurs Rev.

[24] Gishu T, Weldetsadik AY, Tekleab AM (2019). Patients’ perception of quality of nursing care; a tertiary center experience from Ethiopia. BMC Nurs.

[25] Guenther WC (1977). Desk calculation of probabilities for the distribution of the sample correlation coefficient. Am Stat.

[26] Jacobson SF (1997). Evaluating instruments for use in clinical nursing research. Instrum Clin Health Care Res.

[27] Lynn MR, McMillen BJ, Sidani S (2007). Including the provider in the assessment of quality care: development and testing of the Nurses' assessment of quality scale—acute care version. J Nurs Care Qual.

[28] Elayan RM, Ahmad MM (2017). Assessment of the quality of nursing care from perspectives of nurses who experienced hospitalization as patients. J Nurs Care Qual.

[29] Wandelt MA, Ager JW. Quality patient care scale. New York: Appleton-Century-Crofts; 1974. available from: https://scholar.google.com/scholar_lookup?title=Quality%20patient%20care%20scale&publication_year=1974&author=Wandelt%2CMA&author=Ager%2CJW.

[30] Charalambous A, Adamakidou T (2014). Construction and validation of the quality of oncology nursing care scale (QONCS). BMC Nurs.

[31] Palmer Kelly E, Hyer M, Payne N, Pawlik TM (2019). A mixed-methods approach to understanding the role of religion and spirituality in healthcare provider well-being. Psychol Relig Spiritual.

