# Supplementary material for: Development and psychometric testing of quality nursing care scale in Mongolia
Source: BMC Nurs. 2021 Apr 28;20:68. doi: 10.1186/s12912-021-00586-3 (PMC8082924; doi:10.1186/s12912-021-00586-3)
Supplement: Supplementary file 1 — Additional file 1: Appendix A. Number of Study Participants Stratified by Participating Hospital. Appendix B. The Final Version of QNCS-M was shortened from the 66 to 36 items [file 12912_2021_586_MOESM1_ESM.docx]

**Appendix A**

Number of Study Participants Stratified by Participating Hospital

| Name of Hospital | Population | Sample |
| --- | --- | --- |
| First State Central Hospital | 210 | 154 |
| Second State Central Hospital | 168 | 59 |
| Third State Central Hospital | 294 | 138 |
| Khan Uul District Hospital | 52 | 23 |
| Sukhbaatar District Health Center | 51 | 22 |
| Songinokhairkhan District Health Center | 55 | 24 |
| Bayanzurkh District Hospital  Bayangol District Health Center  Chingeltei District Health Center | 55  49  50 | 24  20  21 |
| Total | 984 | 485 |

**Appendix B**

**The Final Version of QNCS-M was shortened from the 66 to 36 items.**

| Five domains from original framework of QNC  (main text page 4,5) | First draft | Number of Items  Deleted by | | | | | Final  version |  |  |
| --- | --- | --- | --- | --- | --- | --- | --- | --- | --- |
|  |  | Content analysis | item-total correlation | Inter-item correlation | Social Desirability | Factor analysis |  | Renamed six domains based  on EFA results.  (main text page 12) | |
| **Physical care**  Symptom management  Activities of daily living  **Psychological care**  Encouragement  Provision of information  **Emotional care**  Empathy  Support  **Social care**  Nurturing relationship  Arranging an environment  **Spiritual care**  Respect for religious beliefs  Concerning culture differences  **Overall QNCS** | 7  9  5  8  7  5  9  5  5  6  66 | 1  3  -  -  -  -  3  -  -  -  7 | 1  -  -  1  -  -  1  -  -  -  3 | 1  -  -  4  1  -  3  1  1  1  12 | -  1  -  1  1  1  -  -  -  2  6 | -  -  -  -  -  1  1  -  -  -  2 | 4  5  5  2  5  3  1  4  4  3  36 | **Independent Nursing Role (4)**  **Interdependent Nursing Role (5)**  **Psychological Element (7)**  **Personal Milieu (9)**  **Social Milieu (4)**  **Spiritual Force (7)**  **Overall QNCS-M (36)** | |
